# Mechanisms of venetoclax resistance and solutions

**DOI:** 10.3389/fonc.2022.1005659

**Published:** 2022-10-12

**Authors:** Jiachen Liu, Yidong Chen, Lihua Yu, Lihua Yang

**Affiliations:** ^1^ The Second School of Clinical Medicine, Southern Medical University, Guangzhou, China; ^2^ Department of Pediatric Hematology, Zhujiang Hospital, Southern Medical University, Guangzhou, China

**Keywords:** venetoclax, resistance, BCL-2 protein, metabolism, oxphos, gene mutations, mitochondria

## Abstract

The BCL-2 inhibitor venetoclax is currently approved for treatment of hematologic diseases and is widely used either as monotherapy or in combination strategies. It has produced promising results in the treatment of refractory or relapsed (R/R) and aged malignant hematologic diseases. However, with clinical use, resistance to venetoclax has emerged. We review the mechanism of reduced dependence on BCL-2 mediated by the upregulation of antiapoptotic proteins other than BCL-2, such as MCL-1 and BCL-XL, which is the primary mechanism of venetoclax resistance, and find that this mechanism is achieved through different pathways in different hematologic diseases. Additionally, this paper also summarizes the current investigations of the mechanisms of venetoclax resistance in terms of altered cellular metabolism, changes in the mitochondrial structure, altered or modified BCL-2 binding domains, and some other aspects; this article also reviews relevant strategies to address these resistance mechanisms.

## Introduction

**Figure 1:**
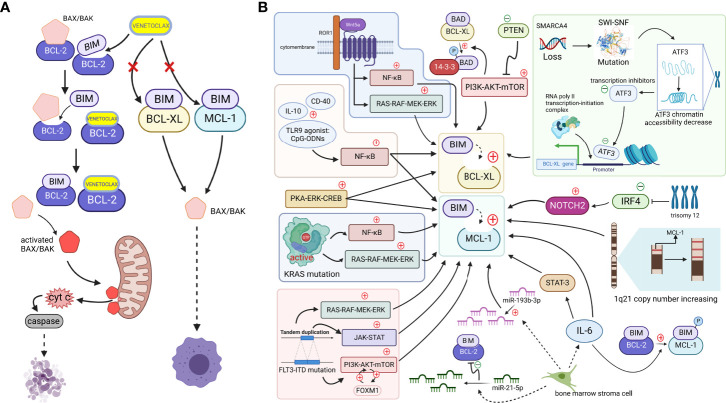
**(A)**. When cells are mainly dependent on BCL-2 for survival, apoptosis is effectively mediated by the action of the small-molecule BCL-2-targeted inhibitor venetoclax. BH3-only proteins are bound to antiapoptotic proteins, ensuring the survival of cancer cells, but venetoclax, a BH3-only protein mimetic, displaces the bound BIM from BCL-2. Free BIM activates the free proapoptotic protein BAX/BAK, and activated BAX/BAK oligomerizes on the outer mitochondrial membrane, forming pore channels and releasing cytochrome c located between the inner and outer mitochondrial membranes into the cytoplasm. Cytochrome c activates caspases in the cytoplasm, stimulating the mitochondrial apoptotic pathway and mediating apoptosis. However, if MCL-1 and BCL-XL expression are upregulated (which may be caused by mechanisms shown in Figure 1b. Since, venetoclax, which only targets BCL-2, does not effectively play a role in releasing the BH3-only protein because non-BCL-2 antiapoptotic proteins bind the BH3-only protein at this time, and thus the cell survives and develops venetoclax resistance (Created with BioRender.com). **(B)**. Tumor cells upregulate other antiapoptotic proteins, such as MCL-1 and BCL-XL, through various signaling pathways or other mechanisms to increase their binding to BH3-only proapoptotic factors, such as BIM. Downregulation of the negative regulator PTEN leads to the activation of the AKT pathway, which directly upregulates the expression of BCL-XL and promotes the dissociation of BAD from BCL-XL and binding to 14-3-3 protein by phosphorylating the BAD protein. The NF-κB pathway, which is activated by microenvironmental agonists such as IL-10, CD40, and TLR9 agonists, and the activated PKA-ERK-CREB pathway induce both BCL-XL and MCL-1 expression, and the MAPK/ERK, PI3K/AKT, and JAK/STAT pathways, with the FOXM1-AKT cycle promoting sustained activation of the AKT pathway. By activating NF-κB and ERK pathways, KRAS mutations or wnt5a-ROR1 signaling pathways can cause upregulated expression of MCL-1 or BCL-XL, respectively. Trisomy 12 CLL cells with low expression of IRF4 upregulate NOTCH2, mediating the high expression of MCL-1. An increased copy number of the MCL-1 locus on chromosome 1q21 might increase MCL-1 expression. SMARCA4 gene deletion, mutations in the SWI-SNF complex and the subsequently reduced chromatin accessibility of the transcriptional repressor ATF3 induce increased expression of BCL-XL. The release of IL-6 from mesenchymal cells directly upregulates the MCL-1 transcript *via* STAT-3. IL-6 promotes BIM phosphorylation, which dissociates BIM from BCL-2 and binds to MCL-1. In addition, reduced miR-193b-3p expression in bone marrow stromal cells mediates MCL-1 upregulation, while upregulation of miR-21-5p reduces BCL-2 levels (Created with BioRender.com).

The abnormal regulation of the apoptosis process is an important part of tumorigenesis. In normal cells, the apoptotic process is strictly regulated by a program involving the BCL-2 family, which is closely associated with hematologic tumors. The BCL-2 family consists of proapoptotic proteins (BAX and BAK), antiapoptotic proteins (BCL-2, BCL-XL, MCL-1, BCL-W, BFL-1, etc.) and BH3-only proteins (BIM, BAD, BID, PUMA, etc.). In surviving normal cells, proapoptotic proteins are bound to antiapoptotic proteins and do not function (1). However, BH3-only proteins release proapoptotic proteins by competitively binding antiapoptotic proteins (2). When the amount of free proapoptotic proteins reach a certain level, the downstream mitochondrial apoptotic pathway is initiated, which causes the proapoptotic proteins to oligomerize and bind to the outer mitochondrial membrane, releasing mitochondrial cytochrome c into the cytoplasm, activating caspases and leading to apoptosis (3). Based on this theory, researchers developed the BH3-only protein mimetic ABT-737 and its derivative navitoclax (ABT-263), which targets three antiapoptotic proteins, BCL-2, BCL-XL, and BCL-W, and releases BH3-only proteins through competitive binding, helping BH3-only proteins promote the release of proapoptotic proteins and activate apoptosis. However, their clinical application is hampered by the toxicity associated with multitarget drugs: navitoclax causes thrombocytopenia in patients before an effective dose is reached (NCT00406809) (4, 5) because its target BCL-2 and BCL-XL, especially the latter, play an important role in platelet survival (6, 7). Due to the lack of progress in multitarget inhibition, scientists have shifted their attention to drugs targeting BCL-2 protein alone for BH3-only protein mimics, because the BCL-2 protein is consistently expressed at high levels in multiple hematologic tumors and is a critical antiapoptotic protein in the BCL-2 family. In 2013, Andrew J Souers et al. used azaindole to replace the indole moiety in the structure of navitoclax and developed the highly selective compound venetoclax (ABT-199) targeting the BCL-2 protein (8). Venetoclax has slight effect on circulating platelet numbers for a potential reason that it is BCL-XL but not BCL-2 playing a critical role in platelet survival (7). As a structural mimic of BH3-only proteins, venetoclax has high affinity for BCL-2 and effectively releases BH3-only proteins. Through competitive binding, BH3-only proteins ultimately inhibit the binding of BCL-2 protein to proapoptotic proteins and induce mitochondria-mediated apoptosis (**Figure 1A**) (9). At present, venetoclax has been reported to be active in several clinical trials (NCT01328626, NCT01794520, NCT01994837) (10–12).

Venetoclax was approved by the FDA for the treatment of chronic lymphocytic leukemia (CLL) in 2016 (13), and it was then gradually applied to research and treatment of other hematologic malignant diseases, such as acute myeloid leukemia (AML) and multiple myeloma (MM). Venetoclax combined with decitabine or azacitidine offers promising responses and survival in older patients with AML (NCT02203773) (14). Venetoclax has also demonstrated efficacy in the treatment of MM (NCT01794520) (11). However, venetoclax based therapies has never been proven to be curative, and its resistance problem must not be ignored: a certain percentage of primary or secondary resistant cells emerge during experiments and the treatment of CLL and AML (15). In this paper, we review current studies on the multiple drug resistance mechanisms of venetoclax in hematologic diseases and the solutions to venetoclax resistance to provide corresponding support, effectively improve the response to drug treatment, and select the appropriate treatment plan.

## Upregulation of the expression of non-BCL-2 antiapoptotic proteins

BCL-2, MCL-1, and BCL-XL, which are members of the BCL-2 family, are all antiapoptotic proteins that promote cell survival and improve the ability of cells to respond to stimuli (16). Their absolute and relative expression profiles may determine the performance of venetoclax to some extent (17). Except for BCL-2 proteins, other antiapoptotic proteins in the BCL-2 family, such as MCL-1 and BCL-XL, are referred to as “non-BCL-2 antiapoptotic proteins” in this review. Recent studies suggest that the upregulation of non-BCL-2 antiapoptotic proteins is the main mechanism of venetoclax resistance. They can substitute the function of BCL-2 when it is bound by venetoclax. This “substitution mechanism” maintains the level of antiapoptotic proteins bound to BH3-only proteins, thereby inhibiting the activation of the mitochondrial apoptotic pathway and resulting in reduced efficacy or even failure of BCL-2-targeted drugs (**Figure 1B**).

Venetoclax shows a high binding specificity for BCL-2, and thus the relative expression levels of the non-BCL-2 antiapoptotic proteins and BCL-2 proteins may be one of the determinants of venetoclax resistance, whether in primary or secondary resistance (18). For example, an earlier study found that cells dependent on MCL-1 or BCL-XL for survival tend to develop primary resistance to venetoclax. Multiple recent studies also revealed that multiple myeloma (MM) cells with higher baseline expression of MCL1 more easily develop venetoclax resistance and show a lower response to even the initial phase of treatment (19–21). After venetoclax treatment, the changes in the relative expression of BCL-2, MCL-1, and BCL-XL, resulted in secondary drug resistance (22, 23). Significantly elevated MCL-1 expression levels were observed in samples collected from patients who relapsed after venetoclax treatment, suggesting that it may be a mechanism to secondary drug resistance (24).

In summary, we propose that one of the strategies to address venetoclax resistance is to design multitarget drugs that simultaneously inhibit multiple antiapoptotic proteins or inhibit them through combination therapy. The combination of low-dose BCL-XL and BCL-W inhibitors, such as navitoclax, with venetoclax may be effective and protects against cellular resistance to venetoclax without dose-limiting thrombocytopenia (NCT03181126) (25, 26). Studies examining the enhanced antitumor effects of MCL-1 inhibitors in combination with BCL-2 inhibitors are also underway (27). However, some studies have also found that multitarget drugs induce toxic effects such as thrombocytopenia (4, 5). Thus, the simple use of multiple proteins as drug targets in an effort to mitigate venetoclax resistance may lead to greater toxicity. The identification of upstream mechanisms regulating antiapoptotic protein expression may deserve consideration.

### Signaling pathways regulating the expression of non-BCL-2 antiapoptotic proteins

In recent years, researchers have identified several signaling pathways that promote the upregulation of non-BCL-2 antiapoptotic proteins such as MCL-1 and BCL-XL.

As a critical signaling pathway involved in cellular life activities, the AKT pathway plays a key role in apoptosis, cell cycle regulation, metabolism, and other processes. Bangyan L Stiles et al. have shown that activation of the AKT pathway upregulates BCL-XL expression and promotes BAD phosphorylation. Phosphorylated BAD shifts the binding from BCL-XL to 14-3-3 proteins. The released BCL-XL protein binds to BAX and BAK to promote antiapoptotic effects (28). Based on the results of a Reverse Phase Protein Array (RPPA) analysis, the investigators suggested that activation of the AKT pathway may be mediated by the downregulation of its negative regulator PTEN (26). In addition, Mikhail S Chesnokov et al. identified the FOXM1-AKT cycle in drug-resistant AML cells, which leads to drug resistance and a poor prognosis for some solid tumors. Although researchers have not directly shown that high FOXM1 expression results in drug resistance, they suggest that the FOXM1-AKT cycle upregulates the MCL-1 protein through sustained activation of the AKT pathway, promoting the development of drug resistance to venetoclax in AML (29).

The NF-κB signaling pathway also is associated with various cellular processes and impacts cellular resistance to venetoclax. Investigators evaluated MCL(mantle cell lymphoma) and CLL cells which were treated with microenvironmental agonists such as IL-10, CD40L and especially the Toll receptor 9 (TLR9)-specific agonist cytosine guanine-oligodeoxynucleotides (CpG-ODNs), and observed a significant increase in the activation of the NF-κB signaling pathway, resulting in drug resistance accompanied by increased expression of BCL-XL and MCL-1 (30).

In secondary resistant MM cells with high expression of MCL-1 and BCL-XL, researchers observed the significant upregulation of genes related to the PKA-ERK-CREB pathway, accompanied by downregulation of apoptosis-related genes. Western blot analysis further confirmed the activation of ERK and its downstream target genes, and the reversal of venetoclax resistance by ERK inhibitors supports the hypothesis that this pathway is involved in drug resistance (31).

In addition to individual pathways, simultaneous activation of multiple pathways leads to drug resistance. Perl A E et al. found FLT3-ITD mutations mediate MCL-1 upregulation by activating the MAPK/ERK, PI3K/AKT, and JAK/STAT pathways in resistant AML cells (32–35). There is also a possible mechanism of upregulation of MCL-1 expression and downregulation of BCL-2 and BAX expression in some AML cells due to activation of NF-KB pathway and ERK pathway by *KRAS* mutations (36). In CLL patients, some of those treated with venetoclax but fail to clear minimal residual disease have high expression of ROR1 and ROR1 expression continues to rise during the development of drug resistance (37). Wnt5a can induce activation of ERK1/2 and enhance CLL-cell proliferation *via* a ROR1/DOCK2-dependent pathway (38). Further study found that Wnt5a-induced ROR1-signaling may enhance expression of ERK1/2 and NF-κB target gene *BCL2L1*, which encoding BCL-XL. These changes may partially contribute to CLL resistance to venetoclax (37).

### Other mechanisms regulating the expression of non-BCL-2 antiapoptotic proteins

Tumor cells promote the upregulation of non-BCL-2 antiapoptotic proteins through numerous other biological processes. Resistance mechanisms have been identified in studies of gene alterations, copy number variations, cytogenetic abnormalities, and the tumor microenvironment.

The upregulation of BCL-XL in mutant MCL cells is associated with SWI-SNF complex mutations produced by *SMARCA4* gene deletion. The protein encoded by the *SMARCA4* gene is an important component of the SWI-SNF complex, which is responsible for chromatin remodeling. The depletion of *SMARCA4* may cause SWI-SNF mutations, leading to abnormal chromatin remodeling and reduced chromatin accessibility in the region encoding the transcriptional repressor ATF3. As a result, ATF3 expression level decreases and the ability of ATF3 to repress the *BCL-XL* transcription loses, which ultimately mediates increased BCL-XL expression (39).

In terms of copy number variation, Maity et al. identified a copy number amplification of the *MCL-1* gene locus (1q21) in MM-resistant cells. Upon relapse, cells with this mutation became the dominant clone of MCL-1 high-expressing cells, suggesting that the elevated MCL-1 expression caused by the increased copy number of the 1q21 locus leads to venetoclax resistance (40).

Cytogenetic abnormalities may lead to the development of drug resistance, which is a finding that has been confirmed in studies of resistance to paclitaxel or cisplatin in patients with gastric cancer (41). Similarly, the study by Fiorcari S et al. highlighted that the development of venetoclax resistance may also be associated with certain cytogenetic abnormalities. They analyzed CLL patient samples and discovered that trisomy 12 subpopulation cells had the unique feature of interferon regulatory factor 4 (IRF4) low expression. The lack of the transcriptional regulator IRF4, a critical regulator of NOTCH signaling pathway, leads to high NOTCH2 expression, which in turn mediates the upregulation of intracellular MCL-1 expression and ultimately leads to the development of primary resistance to venetoclax in trisomy 12 CLL cells. The reduced resistance of cells to venetoclax after knockdown of *NOTCH2* expression further supports this conclusion (42, 43), but the specific relevance of venetoclax resistance to trisomy 12 still requires further exploration.

Tumorigenesis, growth, and metastasis are also inextricably linked to the tumor microenvironment. Esperanza M. Algarín et al. found that the bone marrow stromal cells in tumor microenvironment can decrease the miR-193b-3p expression in MM cells and mediate the upregulation of MCL-1, while a concomitant increase in miR-21-5p expression regulates a decrease in BCL-2 levels. This alteration in the relative ratio of the two proteins contributes to the development of venetoclax resistance (44). Cytokines produced by mesenchymal cells in the microenvironment may also alter the level of dependence of MM cells on the MCL-1 protein. Mesenchymal cells mediate the increase in the dependence of MM cells on MCL-1 by releasing IL-6, which upregulates *MCL-1* transcription in a signal transducer and activator of transcription 3 (STAT3) -dependent manner. Moreover, IL-6 also induces the phosphorylation of BIM protein, which gradually shifts BIM binding from BCL-2 to MCL-1. In the cell line KSM18, IL-6 directly increases MCL-1 transcription through a STAT-3-independent pathway (**Figure 1B**) (45–47).

### Solutions to drug resistance problems

Studies exploring the mechanisms of elevated non-BCL-2 antiapoptotic proteins have provided insights into possible solutions for venetoclax resistance based on signaling pathways. For example, ERK pathway inhibitors help prevent or overcome secondary resistance to venetoclax in patients with MM (31). However, the effect of using inhibitors targeting the AKT pathway alone may be unsatisfactory. Pham LV et al. found that inhibition of the AKT pathway does not sensitize B-cell lymphoma venetoclax-resistant cells with the upregulation of BCL-XL mediated by the AKT pathway, which suggests that treatments targeting the AKT pathway in combination with a BCL-XL inhibitor may be a better therapeutic option. When it comes to the PI3K/AKT pathway, the dual PI3K and HDAC inhibitor CUDC-907 not only overcomes venetoclax resistance by upregulating BIM and downregulating MCL-1 but also synergizes with venetoclax to further promote tumor cell apoptosis because of its DNA damage-mediating effects in AML (48). Rahmani M, Ren W et al. found that the combination of PI3K/mTOR inhibitors (BEZ235, PI-103, etc.) and BCL-2/BCL-XL inhibitors significantly reduces BAX/BAK binding to MCL-1, BCL-2 or BCL-XL in human myeloid leukemia cells, which also indirectly restores the drug treatment effect. This combination significantly inhibits tumor growth and activates tumor cell caspases in a subcutaneous xenograft model, prolonging the survival of mice (49, 50). All of these studies indicate the importance of modulating drug resistance-related signaling pathways to try to overcome venetoclax resistance.

In addition to alterations in signaling pathways, many other factors affecting the upregulation of non-BCL-2 antiapoptotic proteins have provided new ideas to solve this problem. For example, targeting IL-6 or its downstream signaling pathways with anti-IL-6 or IL-6 receptor antibodies may sensitize malignant plasma cells to venetoclax in MM (45). Furthermore, the effect of MEK inhibitors on inhibiting phosphorylation of BIM is to reverse drug resistance (45). For drug resistance in CLL due to MCL-1 upregulation induced by chromatin alterations such as trisomy 12 mutations, the combination of MCL-1 inhibitors with BCL-2 inhibitors produced some results, but further studies are needed to evaluate whether patients’ benefits outweighed the toxicity with these combinations (43). We propose that the development of drugs designed to directly overcome chromosome number alterations, such as drugs that target the chromosome replication process of tumor cells, may be one of the solutions for this mechanism. Although only the addition of the BCL-XL-selective inhibitor a-1331852 has been found to be effective in overcoming resistance related to the SWI-SNF complex mutations in MCL (39), the initiation phase of the mechanism, such as chromatin remodeling, could be targeted, such as the addition of chromatin remodeling-related drugs to venetoclax [e.g., vorinostat and panobinostat (histone deacetylase inhibitors)], a clinical trial of vorinostat in combination with venetoclax in AML is currently underway (NCT05317403). Apart from the two approaches of directly inhibiting *BCL-XL* expression or altering chromatin remodeling, drugs targeting the transcription factor-binding site may also represent a novel treatment.

## Metabolism and cell energy mechanisms

**Figure 2:**
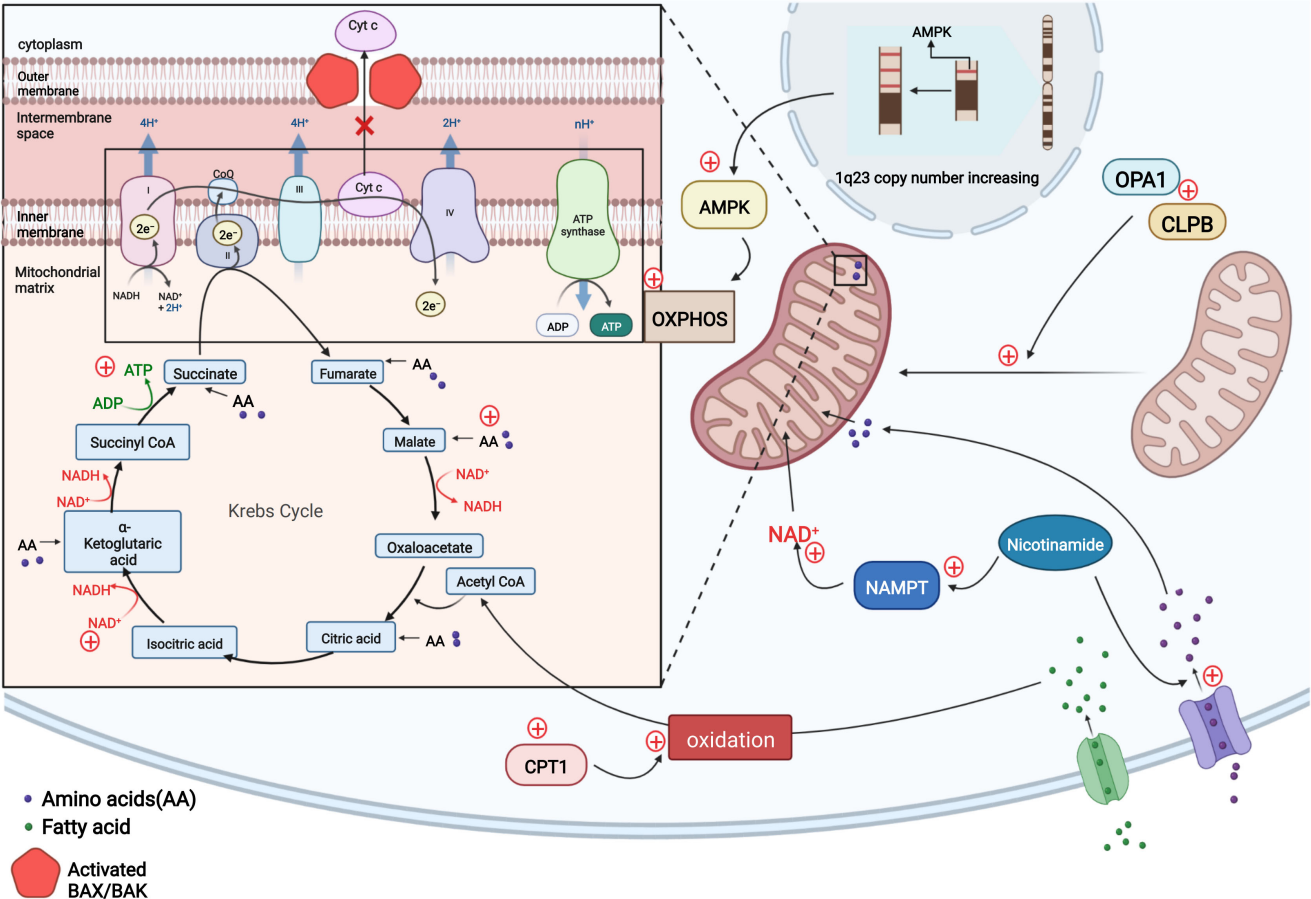
In resistant LSCs, copy number upregulation occurred in the 1q23 chromosome region, leading to the upregulation of AMPK-related proteins and a subsequent increase in the level of OXPHOS to compensate for the impaired energy production induced by venetoclax-mediated cytochrome c release and decreased apoptosis. Nicotinamide activates the TCA cycle and increases ATP levels in two ways by generating NAD+ *via* NAMPT and increasing the levels of TCA intermediates by activating amino acid metabolism. Intracellular CPT1 protein expression is upregulated, and CPT1 promotes fatty acid oxidation to allow fatty acids to enter the TCA cycle and maintain cellular energy. The OPA1 protein and CLPB protein may be upregulated in AML cells to maintain the mitochondrial cristae structure, including increasing the number of mitochondrial cristae and narrowing the cristae lumen to ensure normal mitochondrial function and the inhibition of cytochrome c release. (Adapted from "Electron Transport Chain", by BioRender.com (2020). Retrieved from https://app.biorender.com/biorender-templates).

Cancer cells can develop resistance to venetoclax through metabolism and cell energy mechanisms based on changes in oxidative phosphorylation (OXPHOS), nicotinamide, fatty acid, and glutamine metabolism levels also have been reported (**Figure 2**).

### Increased oxidative phosphorylation levels

Unlike other tumor cells that tend to rely on glycolysis for energy metabolism, AML leukemia stem cells (LSCs) mainly rely on OXPHOS as an energy source (51–53). One of the aspects of the mitochondrial-mediated apoptosis pathway is the release of cytochrome c from mitochondrial intermembrane space, which may indirectly affect cellular OXPHOS levels and may accelerate apoptosis by inhibiting cellular respiration. This data suggests there is tremendous potential of venetoclax in treating hematologic tumors (54). The finding reported by R Guièze et al. that venetoclax rapidly interferes with OXPHOS in sensitive CLL cells also further suggests that venetoclax effectively targets OXPHOS in lymphoid malignancies (24) to promote apoptosis in tumor cells (54). However, the resistance of venetoclax may be caused by the upregulation of OXPHOS. Using oxygen consumption as an indicator to measure the level of mitochondrial electron transport chain (ETC) and OXPHOS, the investigators observed a significant increase in basal and maximum oxygen consumption rates of different kinds of resistant cell lines (treated with venetoclax) compared to sensitive cell lines, indicating an overall increase in the cellular respiration and OXPHOS capacity of resistant cells. Further study revealed that steady-state reactive oxygen species and mitochondrial membrane potential associated with OXPHOS in resistant lines are higher than those in the sensitive. The investigators investigated the factors contributing to these changes and found that the copy number in the chromosomal 1q23 region increased in resistant cells, resulting in the upregulation of AMPK-related proteins encoded in this region, which are key molecules regulating cellular energy metabolism. This change increased OXPHOS levels, and the reduced cellular energy levels caused by venetoclax-induced cytochrome c release are replenished, thereby mediating drug resistance (24, 54). The application of AMPK and ETC complex inhibitors to these cell lines results in increased sensitivity to venetoclax, while activators result in resistance. Similar results were also demonstrated for the combination treatment in mouse experiments. Thus, AMPK activation and mitochondrial metabolic reprogramming can induce drug resistance to venetoclax both *in vitro* and *in vivo*. The investigators concluded that the upregulation of AMPK signaling pathway affects cellular energy, increasing the levels of OXPHOS (54).

### Increased nicotinamide metabolism levels

By comparing the metabolism of refractory or relapsed (R/R) and newly diagnosed AML stem cells, Courtney L. Jones et al. observed significantly elevated levels of nicotinamide and its metabolism in LSCs from R/R patients who are resistant to the combination of venetoclax and azacitidine therapy. By monitoring intermediates of metabolism, they found that resistant cells increase ATP levels by generating NAD+ from nicotinamide through the salvage pathway and increase tricarboxylic acid (TCA) cycle intermediate levels by enhancing amino acid metabolism; both of which promote the TCA cycle. Fatty acid metabolism and glycolysis levels are also increased. All these processes maintain or replace OXPHOS. LSCs, which play a vital role in the development of AML, are preferentially dependent on OXPHOS for energy metabolism (51, 55). Although combination therapy with venetoclax and hypomethylating agents treatment targets nicotinamide metabolism (52, 53), elevated levels of nicotinamide metabolism compensate for the energy gap, allowing cells to become resistant (56).

### Elevated levels of fatty acid metabolism

Oxidized to Acetyl CoA, Fatty acids participate in the TCA cycle and thus maintain OXPHOS. Although venetoclax affects cellular OXPHOS levels to mediate apoptosis, some cells also compensate for this metabolic defect by directly increasing the levels of fatty acid metabolism. During combination therapy of venetoclax and azacitidine of newly diagnosed elderly patients with AML, Brett M. Stevens et al. found that, in addition to elevated nicotinamide metabolism, increased expression of CPT1 protein, which affects β-oxidation of long-chain fatty acids and regulates fatty acid metabolism, was also observed in LSCs, resulting in a significant increase in fatty acid metabolism. More fatty acids enter the TCA cycle and maintain cellular energy levels to compensate for insufficient cellular energy caused by impaired OXPHOS through combination therapy of venetoclax and azacitidine. Knockdown of *CPT1* resulted in decreased fatty acid metabolism levels and reduced compensatory effects on maintaining OXPHOS levels, ultimately leading to a significant decrease in cellular OXPHOS levels, and drug resistance was controlled. In addition to the ability to overcome resistance, inhibition of fatty acid uptake and synthesis enhances the therapeutic effect of the dual BCL-2/BCL-XL inhibitor ABT-737 on AML and sensitizes AML patients to conventional chemotherapy, including cytarabine (57–59).

### Glutamine

Researchers have demonstrated that glutamine inhibition increases both BCL-2 and BIM and therefore mediates increased cellular sensitivity to venetoclax. Furthermore, the investigators hypothesized that increased glutamine levels might inhibit venetoclax. Although the modulation of glutamine metabolism in combination with BCL-2 inhibitors provides better efficacy (60, 61), the specific effects of glutamine inhibition on venetoclax resistance at the molecular level have not been fully characterized.

### Solutions to the drug resistance problem

Hematologic tumor cells have unique metabolic characteristics, such as increased OXPHOS and altered ratios of different energy sources. Drug resistance might be overcome to some extent by hypoxia, caloric restriction (62), and the use of kinase inhibitors (including AMPK) (63) or mitochondrial ETC regulation (64). In addition, earlier studies have shown that the survival of multiple tumor cells, including AML LSCs, is controlled by OXPHOS inhibition (65–68). Thus, the use of drugs targeting OXPHOS in combination with venetoclax is suggested to prevent drug resistance, such as the application of ETC complex inhibitors.

With regard to nicotinamide metabolism, nicotinamide phosphoribosyltransferase (NAMPT) inhibitors effectively restore cellular sensitivity to combination therapy of venetoclax and azacitidine, providing a possible therapeutic strategy (56). NAMPT inhibitors target R/R AML LSCs and have the ability to target different metabolic vulnerabilities in different cancer cells. Essentially, they can inhibit different metabolic pathways, such as glycolysis and OXPHOS, suggesting that they have extensive research potential (56, 65–67, 69, 70). However, at the same time, higher doses of NAMPT inhibitors may be toxic to normal hematopoietic cells, while lower doses do not exert the expected effects on the target LSCs (56). Further clinical evaluations of the dose of NAMPT inhibitors for the selective targeting of LSCs are needed. At this stage, therapeutic options for patients with R/R AML are limited, and at the time of publication of the results of the aforementioned study, no therapeutic strategies were available to directly target stem cells in this patient population. Further studies of NAMPT inhibitors may help fill the gap.

The fatty acid metabolism mechanisms described above also provide rationale to address resistance by combining CPT1 inhibitors with venetoclax. The effects of either knocking down *CPT1* or combining CPT1 inhibitors with venetoclax on acute myeloid leukemia stem cells have been documented *in vitro*, but methods to precisely target abnormally activated CPT1remain to be further explored (57, 59).

## Changes in mitochondrial structure

Maintaining a typical mitochondrial structure is required for cell survival, as it maintains a stable mitochondrial membrane potential, which ensures normal ATP production and mitochondrial homeostasis. Disruption of the membrane potential leads to the release of cytochrome c from the mitochondria to the cytoplasm (71). Venetoclax acts on the mitochondrial cristae structure, causing widening of the cristae lumen and depolarization of the mitochondrial membrane potential (72), promoting the initiation of the mitochondrial apoptotic pathway. Therefore, researchers tried to investigate new mechanisms of venetoclax resistance by observing the alterations in the mitochondrial structure of resistant cells.

### Drug resistance mechanism

Xufeng Chen et al. cultured four venetoclax-resistant AML cell clones using the MOLM-13 and MV4-11 cell lines, and they found that in one of the venetoclax resistant cell lines MV4-11-VR, there was no increase of BCL2 family members (MCL1, BCL2, and BCL-XL), which suggests the existence of alternative modes of resistance acquisition. Electron microscopy revealed that all these cell lines exhibited a narrower and more compact mitochondrial cristae lumen (~14-15 nm in diameter), a greater number of cristae per mitochondrion and higher expression of the OPA1 protein than sensitive parental clones (72). The OPA1 protein acts at the mitochondrial cristae junction and plays a critical role in maintaining cristae stability and preventing the release of cytochrome c (73). With genome-wide CRISPR/CAS9 knockdown technology and RNA sequencing, investigators screened the venetoclax-resistant-related genes and compared them with differentially expressed genes between sensitive and resistant clones, finally obtaining the resistance-associated gene *CLPB*. According to this study, the cristae-forming protein OPA1 maintains the normal structure of mitochondrial cristae by interacting with the mitochondrial chaperone protein CLPB. In the absence of CLPB, OPA1 induces cristae remodeling, the mitochondrial stress response and apoptosis, indicating that CLPB is essential for maintaining the mitochondrial structure and cell survival. Correspondingly, the AML-resistant cell lines treated with venetoclax described above showed a significant increase in CLPB expression, and the cell lines with higher CLPB expression were more resistant to venetoclax. AML cells block the venetoclax-mediated mitochondrial apoptotic pathway by upregulating CLPB to enhance its interaction with the OPA1 protein, thereby stabilizing the mitochondrial structure and inhibiting cytochrome c release. Through RNA sequencing, the investigators also identified genes related to different mitochondrial processes that also displayed altered expression and are related to adaptive changes in mitochondria, such as the regulation of membrane organization and maintenance of the membrane potential. A Gene Ontology (GO) analysis also revealed some changes in the function of mitochondria in resistant cells. They concluded that these changes in the mitochondrial structure are commonly required in venetoclax-resistant AML cells (**Figure 2**) (72).

### Solutions to the drug resistance problem

After knockdown of the *CLPB* gene using sg-RNA, the IC50 values of venetoclax decreased significantly, and the resistant cells regained their sensitivity to venetoclax. An OPA1-specific inhibitor has been investigated to affect mitochondrial function and limit solid tumor growth (74). Further studies are needed to explore whether OPA1 inhibitors play a therapeutic role in hematologic malignancies, including re-sensitizing AML cells to venetoclax.

## BCL-2 binding domain alterations and modifications

Since all BCL-2 family members interact through protein-protein interactions and venetoclax also requires tight binding to antiapoptotic proteins, the affinities between the proteins and venetoclax is essential for venetoclax to function properly. Researchers have identified that tumors alter the drug binding site on the BCL-2 protein through base mutations or epigenetic modifications, freeing the BCL-2 protein from binding to venetoclax and blocking the targeting of the drug.

### Mechanisms of drug resistance

The appearance of point mutations that affect the binding of BCL-2 protein to the BH3 domain is likely to mediate the development of secondary venetoclax resistance, such as the Gly101Val mutation. In CLL, the *BCL-2* Gly101Val mutation (i.e., the substitution of glycine at position 101 of the BCL-2 protein by valine) causes an approximately 180-fold reduction in the affinity of venetoclax for BCL-2 (22). At the same time, this mutation does not influence the affinity of BCL-2 for BIM and therefore apoptosis process is still inhibited (75). In MCL, researchers identified two missense mutations in the same codon of the BH3 domain of the BCL-2 protein in resistant mouse cells (Phe101Cys and Phe101Leu, equivalent to human Phe104Cys and Phe104Leu). The results of immunoprecipitation show that, both mutations weaken the ability of venetoclax to bind to BCL-2 and make the cells resistant (76). In MM cells, another resistance mutation Aps111Ala largely eliminates the venetoclax-induced dissociation of BIM from BCL-2, reducing the sensitivity of the MM-sensitive cell line KMS12PE to venetoclax by approximately 7.5-fold (40). In addition to the mutation described above, Ghia et al. identified a previously unreported nonsynonymous *BCL-2* mutation BCL2A113P, which is located adjacent to the BH3 binding site and is thought to potentially impair the ability of venetoclax to bind and inhibit BCL-2 (37).

**Figure 3:**
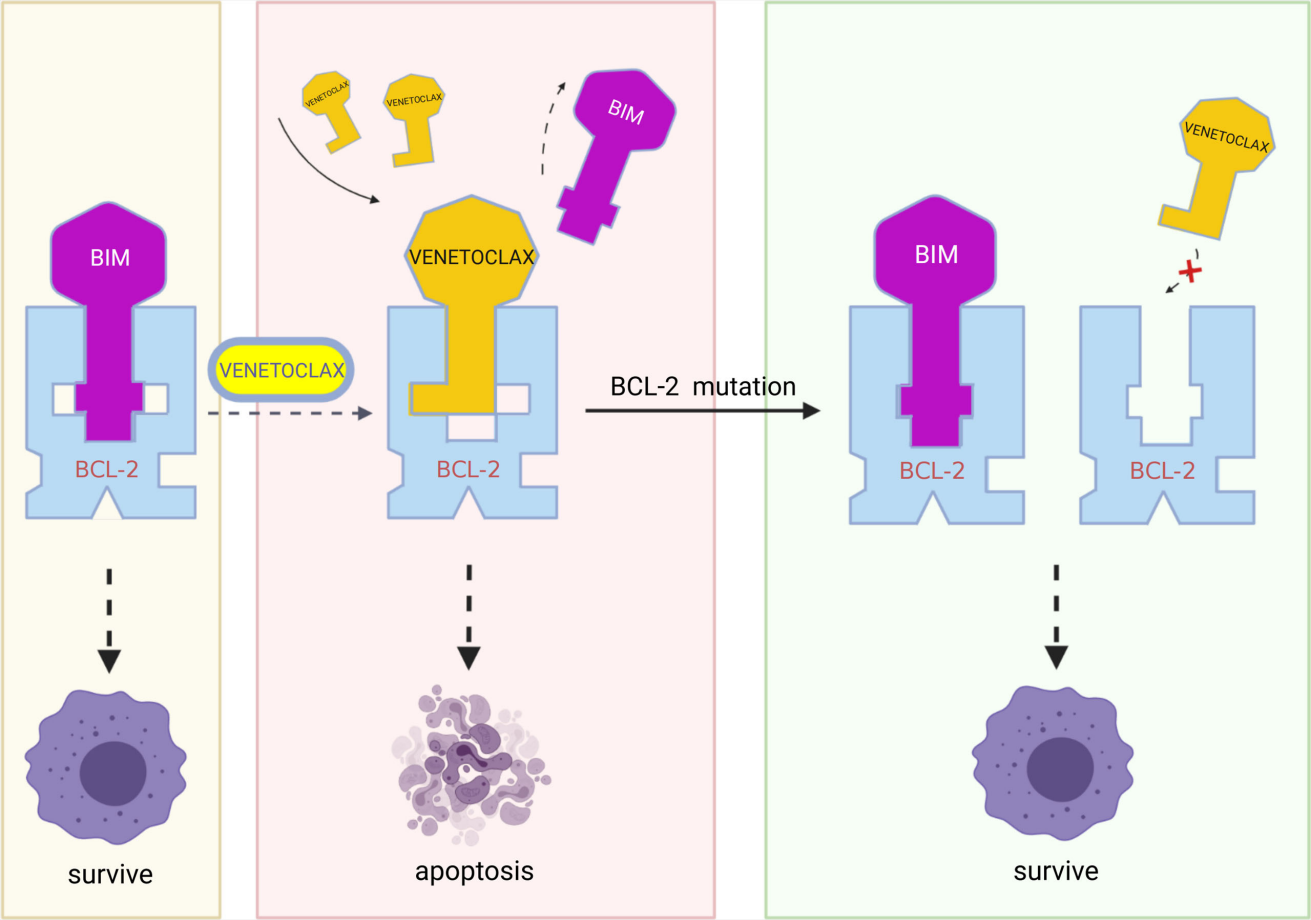
In resistant cells, multiple mutations in BCL-2, such as Gly101Val, Phe101Cys, Phe101Leu, and Aps111Ala, may result in decreased affinity of the BCL-2 protein for venetoclax while retaining the binding of BCL-2 protein to BIM and therefore blocking the action of venetoclax. (Created with BioRender.com.

Besides mutations in BCL-2 binding site, modifications of it also play a role in venetoclax resistance. Researchers have found that though follicular lymphoma expresses BCL-2 at high levels, its response to venetoclax is quite low (77) This result contradicts the viewpoint that BCL-2 protein expression and resistance to venetoclax are negatively correlated. The researchers further found that posttranslational modifications of BCL-2 and its related family members, such as phosphorylation, may prevent venetoclax from displacing BIM on BCL-2, thereby blocking the venetoclax-induced mitochondria-mediated apoptotic pathway (**Figure 3**) (76, 78).

### Solutions to the drug resistance problem

A potential solution to the resistance caused by the decrease in drug-target affinity due to base mutations is unavailable, but researchers have proposed that the Glu152Ala mutation in *BCL-2* rescues the decrease in BCL-2 affinity for venetoclax caused by the Gly101Val mutation in *BCL-2* (75). This phenomenon suggests that further study of the altered spatial conformation of BCL-2 after mutation might be used as a reference for new drug development. Additionally, due to the decrease in drug affinity caused by different mutations, an in-depth study of the change in the 3D structure of BCL-2 upon binding may help solve this problem. For modifications of BCL-2, pBCL-2 kinase inhibitors have been shown to overcome the resistance of CLL cells to ABT compounds (78).

## Other mechanisms and solutions

### Mechanisms of drug resistance

Cells develop resistance to venetoclax through various other mechanisms, such as BAX mutations, RNA methylation, antiapoptotic pathways other than those mediated by the BCL-2 family, tumor suppressor mutations, and abnormal cell cycle regulation.

In CLL patients who have been continuously treated with long-term venetoclax, different mutations in *BAX* may be detected in the myeloid compartment. Some of them occur in the critical BAX α9-helix which targets BAX to the mitochondrial outer membrane. These α9-helix mutations may fail venetoclax to induce apoptosis. The investigators re-expressed several α9-helix mutations in the AML cell line MOLM-13 and found these AML cells also exhibited significant venetoclax resistance. These results suggest that *BAX* mutations may serve as a resistance mechanism for AML and CLL (79).

The RNA cytosine methyltransferases NSUN2 and NSUN1 modify the methylation of cytosine in RNA and have functions such as stabilizing tRNA and mRNA. In leukemia cells, NSUN2 and NSUN1 alter the RNA transcription process as part of the RNA polymerase II extension complex (eRNAPII), causing cells to develop resistance to venetoclax. Venetoclax strongly induces the expression of eRNAPII and NSUN1 to convert sensitive cells into resistant cells, and the expression levels of eRNAPII, NSUN2, and NSUN1 are strongly correlated with the degree of lineage-associated resistance to venetoclax. After knocking down *NSUN1* or *NSUN2* by siRNAs, venetoclax-resistant K1VR lineage leukemia cells resensitize to low doses of venetoclax (80).

The growth arrest-specific protein 6 (GAS6)/AXL axis is upregulated in various tumor tissues, and it has been suggested to be another antiapoptotic pathway in addition to the BCL-2 family in some tumors (81–83). Upon binding to AXL, GAS6 activates downstream signaling pathways and promotes tumor cell survival, proliferation, migration, and other activities (81, 84, 85). GAS6/AXL axis correlates with the expression of several leukemic stem cell marker genes, and increased AXL and GAS6 expression is associated with poor prognosis for individuals with AML (86–88). Based on these findings, LSCs may develop venetoclax resistance by increasing the activity of the GAS6/AXL axis to partially counteract the proapoptotic effect produced by venetoclax by acting on BCL-2 family-related proteins.

In addition, AML also exhibits resistance mechanisms mediated by mutations in the tumor suppressor gene *TP53*. Full TP53 function is critical for BH3-mimetic drugs efficacy, and if *TP53*-mutant clones emerge under the therapeutic pressure of sublethal doses of BCL-2 or MCL-1 inhibitors, drug resistance will occur (89). Furthermore, another study found that biallelic mutations of *TP53* are associated with primary drug resistance, especially for venetoclax combined with azacitidine or cytarabine (90).

Cell cycle alterations are also a major feature of carcinogenesis, and tumor cells often sustain proliferation for abnormal cell cycle checkpoints. For example, a single-cell transcriptome analysis of a cohort of patients with MCL found that the G2/M checkpoint protein was significantly upregulated in ibrutinib-venetoclax-resistant patients and was strongly correlated with disease progression (91, 92). Another study on MM found that the expression of the *G0S2* gene, which mediates the transition of cells from the G0 to G1 phase, was significantly decreased in drug-resistant cell lines compared to sensitive lines. Further exploration of the mechanism revealed that G0S2 inhibited the BCL-2 protein, and the downregulation of G0S2 may lead to an increase in BCL-2 expression (31).

Another finding in the studies of drug resistance deserves our attention—the spatial heterogeneity of resistant leukemia cells. A study examined the differences in protein expression in resistant cells of different locations by continuously monitoring CLL cells in the bone marrow and peripheral blood. The expression of X-linked apoptosis inhibitory protein (XIAP) was significantly higher in resistant cells in bone marrow than in peripheral blood before venetoclax treatment (day 0) and acquired resistance status (day 450), and BCL-2 was expressed at higher levels in peripheral blood. This result may suggest that CLL cells at different locations employ different resistance mechanisms (93). According to previous studies, elevated XIAP levels may result from the activity of the AKT pathway (94), suggesting that CLL cells in the bone marrow may have elevated XIAP levels through the activation of the AKT pathway in response to venetoclax, mediating drug resistance. In contrast, this mechanism only exerts a relatively minor effect on CLL cells in peripheral blood.

### Solutions to drug resistance problems

Recent epigenetic studies have reported that DNA methylation inhibitors, histone deacetylase inhibitors, histone methyltransferase EZH2 inhibitors, and bromodomain inhibitors respectively act in combination with venetoclax by modulating epigenetic mechanisms, rendering cells more sensitive to venetoclax (95–100). Moreover, PLK1 plays a critical role in regulating the G2/M transition of the cell cycle by phosphorylating PTEN to regulate the PI3K-AKT pathway, and ultimately affects the G2/M transition. The PLK1 inhibitor volasertib, which significantly arrests MCL tumor cells at the G2/M transition, has been documented to recover cellular sensitivity to venetoclax and ibrutinib when administered in combination with the PI3K inhibitor copanlisib (92).

Additionally, Xiaojia Niu et al. revealed excellent synergistic effects of the combination of the GAS6/AXL axis inhibitor SLC-391 with venetoclax treatment on inhibiting AML stem/progenitor cell proliferation and long-term clonal activity. The levels of PARP and caspase-3, which play key roles in apoptosis, were shown to increase in the combination treatment group, and the combination of the two drugs was effective in regulating the cell cycle by inducing an increase in the number of G1 phase cells to promote apoptosis. By exploring the specific mechanism, researchers found that SLC-391 combined with venetoclax selectively targets AML stem/progenitor cells. They effectively regulate OXPHOS by downregulating AXL signaling proteins and reducing the mitochondrial membrane potential in CD34^+^ AML stem/progenitor cells. They also inhibit the MEK-ERK pathway and PI3K-AKT pathway. These changes interfere with cellular metabolism in several ways to kill stem/progenitor-resistant cells synergistically. This combination strategy exerts a better effect on killing AML cells with high expression of GAS6/AXL (101).

SF3B1 functions as an essential part of spliceosome assembly and its mutations can be seen in many hematologic malignancies (102). Based on this, the researchers found that splicing inhibitors can sensitize multiple myeloma cells to venetoclax through its affection on MCL-1 transcripts, shifting cells from MCL-1-dependent to BCL-2-dependent (103).

Recently, researchers reported that triggering an accumulation of ceramide by inhibition of sphingosine kinase induced an apoptotic integrated stress response in AML cells, leading to transcription of the BH3-only protein NOXA and degradation of the prosurvival MCL-1 protein, which may overcome BCL-2 inhibitor resistance (104).

## Early warning for resistance events by detecting drug resistance-related specific signatures

Along with the continuous research on drug resistance mechanisms, researchers have begun to search for signatures that predict the sensitivity of tumor cells to venetoclax before treatment or monitor resistance during treatment to select or switch to appropriate drugs as early as possible and help prolong patient survival.

The development of primary drug resistance is effectively predicted by the expression levels of certain markers. AML cell surface markers CLEC7A (CD369) and CD14 positively correlated with the AUC of venetoclax (r=0.68 and 0.64, p<0.0001), in other words, patients with AML presenting high CD14/CLEC7A expression exhibited reduced sensitivity to venetoclax monotherapy, suggesting their potential in predicting venetoclax resistance (105). In addition to cell surface markers, the expression levels of the GAS6/AXL axis in cells have been shown to reflect primary resistance to venetoclax in AML cells and can be used as a marker to predict response to therapy, as well as the potential to indicate the need for combination therapy with AXL/BCL-2 inhibitors (101). Another more approaches to predict primary resistance in addition to measuring expression levels is detecting the mutations of genes or variations of chromosome number. For example, the detection of *FLT3*-ITD mutations in AML cells may predict their response to venetoclax, and combining the FLT3 inhibitor quizartinib (106) or gilteritinib (32, 107) with venetoclax may result in a better therapeutic effect. Clinical trials have also been conducted in combination with gilteritinib (NCT03625505). In addition, CLL cells with trisomy 12 potentially develop primary resistance to venetoclax due to specific alterations in the IRF4/NOTCH2 axis, and the initial treatment phase has been unsatisfactory (42, 43), suggesting that we should choose another appropriate drug if trisomy 12 of CLL cells occurs.

Moreover, some monitoring methods have also been explored for secondary drug resistance developed during treatment. Shanshan Pei et al. found that resistant monocytes (CD45^-^bright/SSC-high/CD117^-^/CD11b^+^/CD68^+^) differentiated after drug treatment showed a significantly different response from typical FAB-M0/M1/M2 bone marrow cells (CD45^-^med/SSC-low/CD117^+^/CD11b^-^/CD68^-^) in AML patients. Patients with the latter immunophenotype may achieve complete remission (CR) with combination therapy of venetoclax and azacitidine, and patients with the former immunophenotype may have no response to it (108). From M0 to M5, the level of MCL-1 was progressively increasing and the level of BCL-2 was decreasing. Patients with preexisting monocyte subclones, after combination therapy of venetoclax and azacitidine, appeared to show striking *in vivo* selection for the monocytic subpopulation (109). Based on these results, we can monitor the development of resistant monocyte differentiation by detecting the cellular immunophenotype during the treatment and then adjust the appropriate treatment regimen to avoid refractory relapse as much as possible. In addition to detecting the cellular immunophenotype, the copy number variation in the 1q21 region of the *MCL-1* gene also predicts the sensitivity of cells to venetoclax. If amplification of the 1q21 region in MM cells is detected during treatment, it often suggests elevated MCL-1 expression and the development of resistance (40). In metabolic studies, the level of fatty acid metabolism was reported to correlate significantly with the patient response to combination therapy of venetoclax and azacitidine, and increased fatty acid metabolism during treatment suggested drug resistance (57). Additionally, the expression level of the mitochondria-associated protein CLPB was also positively correlated with the degree of AML resistance and may indicate secondary drug resistance in cell lines (72). However, due to the small statistical sample size of the cell lines, this conclusion requires further confirmation.

## Conclusions

Venetoclax, a novel small-molecule targeted BCL-2 inhibitor, has fewer side effects than multitarget drugs such as ABT-737, effectively enhancing the therapeutic utility of BCL-2 inhibitors and filling part of the therapeutic gap. Combination therapy based on venetoclax may provide a delayed recurrence or even remission in some patients with R/R hematological malignant diseases (110–112). However, resistance to venetoclax also exists. Malignant cells directly antagonize the effects of venetoclax by upregulating the expression of non-BCL-2 antiapoptotic proteins in the BCL-2 family to replace BCL-2 and continuing to maintain binding to BIM proteins. This “substitution mechanism” is also how cells classically develop venetoclax resistance.

In addition, in cellular metabolism, alterations in the level of OXPHOS and changes in the metabolism of nicotinamide or fatty acids help cells develop resistance. In particular, an increased level of fatty acid metabolism is a general mechanism for developing resistance to chemotherapeutic drugs (113–115). Thus, the continued investigation of the molecular mechanisms of fatty acid metabolism leading to drug resistance and the identification of targeted therapeutic might be beneficial to more targeted tumor therapies. Glutamine metabolism also affects the cellular response to venetoclax, but the specific mechanisms of drug resistance require further investigation. Direct changes in the structure of mitochondrial cristae are potentially involved in the development of cellular drug resistance. Moreover, multiple mutated forms of the *BCL-2* gene also contribute to drug resistance by reducing the affinity of the BCL-2 protein for venetoclax.

On the other hand, the effect of epigenetic alterations on tumorigenesis is receiving increasing attention. Researches have reported the development of venetoclax resistance by mediating alterations in methylation during RNA transcription. Cell cycle, alterations in tumor suppressor gene expression, and effects of other processes on venetoclax resistance are all being investigated.

As resistance mechanisms are being explored, the solutions to some of these mechanisms are also being assessed. Signaling pathway inhibitors such as CUDU-907 and BEZ235, cell metabolism-related drugs such as AMPK inhibitors and NAMPT inhibitors, epigenetic-related drugs such as DNA methylation inhibitors, histone deacetylase inhibitors, and cell cycle regulators like volasertib, and the GAS6/AXL axis inhibitor SLC-391 all work well in combination with venetoclax to exert a significant synergistic effect.

With the ongoing investigation of drug resistance mechanisms and the discovery of relevant biomarkers, we expect to be able to detect different mechanisms of cancer development and drug resistance in different patients using more precise methods to finally achieve precise individualized treatment and help more patients achieve remission and return to good health.

## Author contributions

JL and YC collected the related paper and finished the manuscript and figures. LYu revised the article. LYa gave constructive guidance and made critical revisions. All authors read and approved the final manuscript.

## Conflict of interest

The authors declare that the research was conducted in the absence of any commercial or financial relationships that could be construed as a potential conflict of interest.

## Publisher’s note

All claims expressed in this article are solely those of the authors and do not necessarily represent those of their affiliated organizations, or those of the publisher, the editors and the reviewers. Any product that may be evaluated in this article, or claim that may be made by its manufacturer, is not guaranteed or endorsed by the publisher.

## Glossary

**Table:**

BCL-2	(B-cell lymphoma-2)
OXPHOS	(oxidative phosphorylation)
MCL-1	(myeloid cell leukemia-1)
BCL-XL	(BCL2 like 1)
BCL-W	(BCL2 like 2)
BFL-1	(BCL2 related protein A1)
BAX	(BCL2-Associated X)
BAK	(BCL2 antagonist/killer 1)
BIM	(BCL2 like 11)
BAD	(BCL2 associated agonist of cell death)
BID	(BH3 interacting domain death agonist)
PUMA	(BCL2 binding component 3)
CLL	(chronic lymphocytic leukemia)
AML	(acute myeloid leukemia)
MM	(multiple myeloma)
MCL	(mantle cell lymphoma)
RPPA	(Reverse Phase Protein Array)
PTEN	(phosphatase and tensin homolog)
FOXM1	(forkhead box M1)
NF-κB	(nuclear factor kappa-B)
TLR9	(Toll receptor 9)
CpG-ODNs	(Cytidine-phosphatte-guanonine Oligodeoxynucleotides)
PKA	(Protein Kinase A)
CREB	(cAMP-response element binding protein)
MEK	(mitogen-activated protein kinase kinase)
ERK	(extracellular regulated protein kinase)
FLT3-ITD	(fms related receptor tyrosine kinase-internal tandem duplication)
MAPK	(mitogen-activated protein kinase)
PI3K	(phosphoinositide 3-kinase)
JAK	(Janus kinase)
SWI-SNF	(Switch/Sucrose Non-fermentable)
SMARCA4	(SWI/SNF related matrix associated actin dependent regulator of chromatin subfamily a member 4)
ATF3	(activating transcription factor 3)
IRF4	(interferon regulatory factor 4)
STAT	(signal transducer and activator of transcription)
IL-6	(interleukin 6)
HDAC	(histone deacetylase)
mTOR	(mammalian target of rapamycin)
LSCs	(leukemia stem cells)
ETC	(electron transport chain)
AMPK	(Adenosine 5&rsquo;-monophosphate (AMP)-activated protein kinase)
TCA	(tricarboxylic acid)
CPT1	(Carnitine palmitoyltransferase 1)
NAMPT	(nicotinamide phosphoribosyltransferase)
R/R	(relapsed/refractory)
CLPB	(caseinolytic mitochondrial matrix peptidase chaperone subunit B)
OPA1	(Optic Atrophy 1)
GO analysis	(Gene Ontology analysis)
eRNAPII	(RNA polymerase II extension complex)
GAS6	(growth arrest-specific protein 6)
TP53	(tumor protein p53)
XIAP	(X-linked apoptosis inhibitory protein)
EZH2	(enhancer of zeste 2 polycomb repressive complex 2 subunit)
PLK1	(polo like kinase 1)
PARP	(poly (ADP-ribose) polymerase 1)
CLEC7A	(C-type lectin domain containing 7A)
AUC	(Area under Curve)
CR	(complete remission)
